# Supplementary material for: Resolving the full spectrum of human genome variation using Linked-Reads
Source: Genome Res. 2019 Apr;29(4):635–45. doi: 10.1101/gr.234443.118 (PMC6442396; doi:10.1101/gr.234443.118)
Supplement: Supplemental Material [file supp_gr.234443.118_Supplemental_longranger-2.2.zip › longranger-2.2.tar.bz2!/lr2.2-public/lib/stan/cmdstan/stan/doc/api/html/namespacemembers_type.html]

Stan: Namespace Members


|  |  |
| --- | --- |
|  | Stan  2.5.0  probability, sampling & optimization |


- Main Page
- Related Pages
- Namespaces
- Classes
- Files

- Namespace List
- Namespace Members

- All
- Functions
- Variables
- Typedefs
- Enumerations
- Enumerator

- b
- f
- i
- m
- p
- r
- s
- u
- v


All Classes Namespaces Files Functions Variables Typedefs Enumerations Enumerator Friends Macros Pages

### - b -

- base\_expr\_type
  : stan::gm
- bool\_argument
  : stan::gm

### - f -

- function\_signature\_t
  : stan::gm

### - i -

- input\_iterator\_t
  : stan::gm
- int\_argument
  : stan::gm

### - m -

- matrix\_d
  : stan::math
  , stan::optimization
- matrix\_fd
  : stan::agrad
- matrix\_ffd
  : stan::agrad
- matrix\_ffv
  : stan::agrad
- matrix\_fv
  : stan::agrad
- matrix\_v
  : stan::agrad

### - p -

- pos\_iterator\_t
  : stan::gm

### - r -

- real\_argument
  : stan::gm
- row\_vector\_d
  : stan::math
- row\_vector\_fd
  : stan::agrad
- row\_vector\_ffd
  : stan::agrad
- row\_vector\_ffv
  : stan::agrad
- row\_vector\_fv
  : stan::agrad
- row\_vector\_v
  : stan::agrad

### - s -

- size\_type
  : stan::agrad
  , stan::math
- string\_argument
  : stan::gm

### - u -

- u\_int\_argument
  : stan::gm

### - v -

- var\_origin
  : stan::gm
- vector\_d
  : stan::math
  , stan::optimization
- vector\_fd
  : stan::agrad
- vector\_ffd
  : stan::agrad
- vector\_ffv
  : stan::agrad
- vector\_fv
  : stan::agrad
- vector\_v
  : stan::agrad


---

[ Stan Home Page ]

*© 2011–2014,
Stan Development Team.*
